# From Resources to Beliefs: The Mediating Roles of Parental Educational Involvement in the Association Between Parental Psychological Capital and Adolescents’ Academic Self-Efficacy in Shanxi Province, China

**DOI:** 10.3390/bs16091525

**Published:** 2026-08-29

**Authors:** Ting Zhao, Asmah Ismail, Engku Mardiah Engku Kamarudin

**Affiliations:** 1 Faculty of Educational Studies, Universiti Putra Malaysia (UPM), Serdang 43400, Selangor, Malaysia; gs64191@student.upm.edu.my (T.Z.); engkumardiah@upm.edu.my (E.M.E.K.); 2 Department of Physics and Electronic Engineering, Yuncheng University, Yuncheng 044000, China

**Keywords:** academic self-efficacy, parental psychological capital, parental educational involvement, mediating roles

## Abstract

Parental psychological resources are important factors that may impact the academic self-efficacy of adolescents. Drawing on family systems, social cognitive, and psychological capital theories, this study employed a questionnaire survey design to examine the associations between adolescents’ perceived parental psychological capital and their academic self-efficacy and to investigate the mediating role of perceived parental educational involvement. Furthermore, father- and mother-related pathways were compared to identify potential differences in both direct and indirect effects. Validated questionnaires were completed by a total of 472 students, and structural equation models were estimated separately for father- and mother-related pathways. Differences in direct effects were examined using chi-square difference tests between constrained and unconstrained models, while differences in indirect effects were assessed using bootstrapped confidence intervals. The results showed that both perceived father’s psychological capital and mother’s psychological capital were positively associated with adolescents’ academic self-efficacy, with paternal and maternal educational involvement partially mediating these associations. Comparative analyses showed that the association between mothers’ educational involvement and adolescents’ academic self-efficacy was stronger than that between fathers’ educational involvement and adolescents’ academic self-efficacy, whereas no significant difference was found between father- and mother-related psychological capital pathways. In addition, the indirect effect through mothers’ educational involvement was significantly stronger than that through fathers’ educational involvement, suggesting differential parental pathways in adolescents’ academic development. These findings highlight the importance of adolescents’ perceptions of parental psychological resources and educational involvement in promoting academic confidence and motivation, providing practical implications for parent-focused interventions.

## 1. Introduction

Academic self-efficacy (ASE), which is described as a student’s beliefs in their abilities to organize and complete learning tasks (Bandura, 1977), is one of the most important psychological factors related to academic motivation, persistence, learning engagement, and academic achievement (Honicke & Broadbent, 2016; Jinks & Morgan, 1999). Early adolescence (roughly junior high school age) is a key developmental period where academic requirements increase and psychological and social development undergo rapid changes. ASE is especially malleable and sensitive to contextual influences during this period of time (Gebauer et al., 2020; Llorca et al., 2017).

In this developmental process, the family is among the most important environments that influence adolescents’ academic beliefs. In recent years, family education and students’ psychological development have been increasingly highlighted in educational reform and policy in China (General Office of the State Council of the People’s Republic of China, 2021; Ministry of Education of the People’s Republic of China et al., 2023). It is important to shift from a narrow view on educational outcomes to a more extensive view that considers the psychological and behavioral mechanisms of the family as factors influencing students’ internal motivational resources, with academic self-efficacy as a central factor (Shao & Kang, 2022).

### 1.1. Parental Psychological Capital, Parental Educational Involvement, and Academic Self-Efficacy

Hope, efficacy, resilience, and optimism are all considered positive psychological resources that make up psychological capital, which has been shown to be a strong predictor of adaptive functioning and performance in achievement-related contexts (Luthans et al., 2007; Luthans & Youssef-Morgan, 2017). Parental psychological capital (PPC) is defined as parents’ ability to cope with the demands of education, control stress, and promote their children’s growth in the family setting (Cole & Molloy, 2023; Yue et al., 2023).

Parental educational involvement (PEI) is defined as parents’ participation in their children’s learning activities, such as supervising their learning, communicating regarding school matters, and providing learning-related support (Epstein, 2011; Hill & Tyson, 2009). Previous studies have suggested that such participation depends not only on structural factors such as time and socioeconomic assets but also on the parents’ internal psychological factors (Grolnick & Slowiaczek, 1994; Tazouti & Jarlégan, 2019).

Adolescents’ ASE is a developmental process that is influenced by their interactions with significant others and by how they cognitively interpret these interactions (Bandura, 1986). Parents, as the closest social actors in adolescents’ academic worlds, can present significant efficacy-related messages through their beliefs, emotion regulation, and coping strategies in educational settings (Pajares, 1996; Schunk & Pajares, 2009). PPC, which comprises hope, self-efficacy, resilience, and optimism, may therefore serve as an important source of efficacy-related information that adolescents internalize in the development of their academic self-efficacy.

According to family system theory, the family is a mutually dependent system, where the psychological resources of the parents and the development of the adolescents are constant relational transactions. While there is limited direct empirical evidence linking PPC to adolescents’ ASE, there is indirect evidence through PPC’s key elements. Parental self-efficacy has attracted the most interest because it plays an essential role in maintaining family well-being and supporting child development (Albanese et al., 2019; De Jong et al., 2022). Moreover, parents’ hope and optimism contribute to a positive emotional atmosphere that fosters adolescents’ positive beliefs (Cole & Molloy, 2023; Pratt et al., 2001), and parental resilience serves as a protective factor that influences the emotional climate of the family and consequently affects adolescents’ coping strategies through modeling and emotional transmission (Gavidia-payne et al., 2015). According to these dimensions, psychological capital is considered an integrative construct that enhances parenting competence and is seen as a vital family asset (Yue et al., 2023; Yuan & Yue, 2022).

According to psychological capital theory, individuals with high levels of hope, self-efficacy, resilience, and optimism are more likely to persevere, face obstacles, and stay engaged in difficult work (Luthans et al., 2007). From a parenting perspective, educational involvement involves continuous emotional investment and handling of students’ academic strains, which indicates that parents’ psychological resources play an important role in their educational involvement. While little research has directly linked parental psychological capital and educational involvement, studies have mostly focused on related constructs, with parental psychological capital being found to be positively associated with parenting competence (Yue et al., 2023). There is a clear and consistent link between parental self-efficacy, as well as involvement in child learning, and closer parent–school connections (Ma et al., 2024). In general, the current evidence points indirectly to PPC as a precursor of PEI.

According to social cognitive theory, learning through mastery experiences, verbal persuasion, and social modeling are key mechanisms in the process of acquiring ASE (Bandura, 1997). PEI supports these processes by providing structure for the learning environment, support for adolescents’ academic work, and parental expectations and confidence (Pajares, 1996). A significant number of empirical studies have revealed that there is a positive association between increases in PEI and adolescents’ ASE (Fan & Williams, 2010; Lv et al., 2018; Zhao et al., 2025). This association may be especially relevant during junior high school, when there is a tendency for greater independence and sensitivity to parental support (De Los Reyes et al., 2019). Overall, the findings highlight the value of PEI as a proximal influence on adolescents’ ASE.

However, the literature is still lacking in some areas. There is a dearth of empirical work directly connecting PPC and adolescents’ ASE, especially in early adolescence in China. There is little research on the combination of PPC and PEI, including the distinction between fathers and mothers. The present study emphasizes the inter-connections between PPC, PEI, and adolescents’ ASE by drawing on family systems, social cognitive, and psychological capital theories. The present study proposes the following hypotheses:

**H1a.**  *Adolescents’ perceptions of FPC are positively associated with their ASE*.

**H1b.**  *Adolescents’ perceptions of MPC are positively associated with their ASE*.

**H2a.**  *Adolescents’ perceptions of FPC are positively associated with FEduI*.

**H2b.**  *Adolescents’ perceptions of MPC are positively associated with MEduI*.

**H3a.**  *Adolescents’ perceptions of FEduI are positively associated with their ASE*.

**H3b.**  *Adolescents’ perceptions of MEduI are positively associated with their ASE*.

### 1.2. The Mediating Effect of Parental Educational Involvement

By combining psychological capital and social cognitive theories, it is considered that PEI may play a role in the relationship between PPC and adolescents’ ASE; however, empirical evidence for this pathway is still scarce. While PPC reflects parents’ internal psychological resources, educational involvement represents a concrete behavioral manifestation of these resources within the family system. Empirical investigations have provided initial evidence supporting the mediating role of PEI in the relationship between parental psychological attributes and students’ academic performance (Tazouti & Jarlégan, 2019). Nevertheless, relatively few studies have directly explored this mediating mechanism from the perspective of students, particularly within junior high school populations (De Los Reyes et al., 2019). Moreover, potential differences in mediation pathways between fathers and mothers remain insufficiently explored (Kim & Hill, 2015).

Consequently, this study posits a mediating model wherein PEI bridges perceived PPC and junior high school students’ ASE, with separate mediation effects for fathers and mothers. Based on this, this study puts forward the following hypotheses:

**H4a.**  *Adolescents’ perceived FEduI mediates the relationship between perceived FPC and their ASE*.

**H4b.**  *Adolescents’ perceived MEduI mediates the relationship between perceived MPC and their ASE*.

### 1.3. Father–Mother Comparative Models

Family system theory focuses on the differentiated but related roles of parents in the family system (Bowen, 1978). Differences between fathers and mothers in terms of psychological traits, educational involvement styles, and interaction styles with adolescents may lead to different mechanisms of influence on adolescents’ academic self-efficacy (Kim & Hill, 2015; Liu et al., 2022).

Although a growing body of work acknowledges these distinctions, empirical studies often conceptualize parental effects as undifferentiated, and there is limited evidence of nuanced father and mother effects (Kim & Hill, 2015). Separate analyses of father- and mother-based models enable a more precise analysis of whether the structural relationships between PPC, PEI, and ASE vary according to parent gender (Liu et al., 2022). Following this, the present study tests for multi-group structural disparities between father- and mother-based models in terms of the direct and indirect pathways.

Based on this, this study proposes the following hypotheses:

**H5a.**  *The structural relationships among adolescents’ perceived PPC, PEI, and ASE differ significantly between father- and mother-based models*.

**H5b.**  *The mediating effect of adolescents’ perceived PEI on the relationship between perceived PPC and ASE differs significantly between father- and mother-based models*.

**Figure 1:**
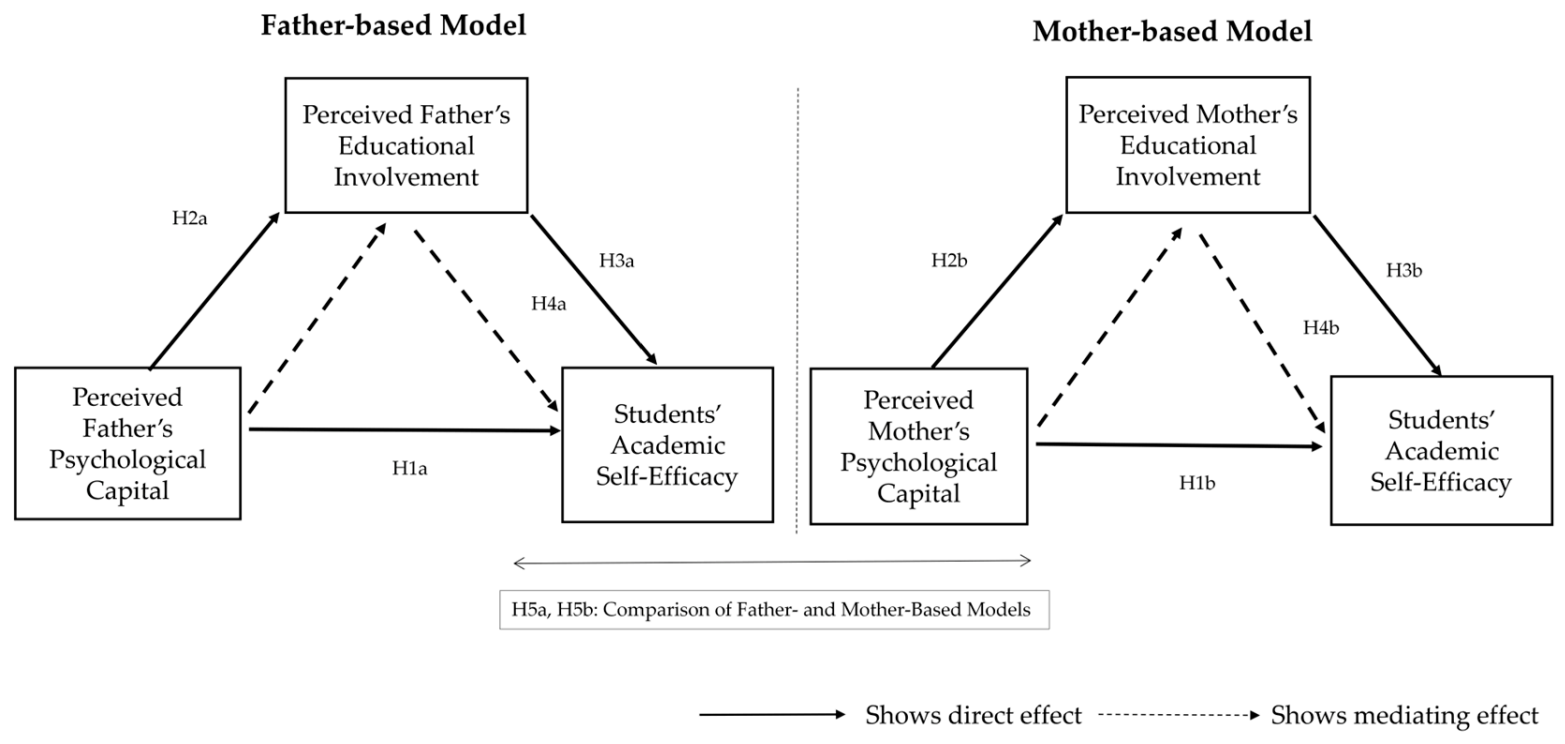
The conceptual framework of this study.

In this study, family systems, social cognitive, and psychological capital theories are combined to construct mediation models for both the father and mother pathways. This study aims to examine the influence of PPC on junior high school students’ ASE, explore the role of perceived PEI, and systematically compare the direct and mediating effects between father and mother models (see Figure 1).

## 2. Materials and Methods

### 2.1. Participants and Procedures

This study employed a cross-sectional survey design. The study procedures were approved by the university’s ethics board and conducted in accordance with relevant ethical standards (Approval No. JKEUPM-2025-025). After ethical approval was obtained, written informed consent was obtained from the relevant educational institutions, teachers, and parents of the participating students. Participants were informed that their participation in this investigation was entirely voluntary and that they could withdraw at any time. A two-stage stratified random sampling strategy was employed to obtain the study sample. In the first stage, the study area was stratified into urban, town, and rural areas to ensure representation of different geographical contexts, followed by the random selection of one school from each stratum. In the second stage, student lists provided by the selected schools were used as the sampling frame, and eligible enrolled students in Grades 7–9 were randomly selected from these lists. The questionnaires were administered in paper form in classrooms and completed under the supervision of trained researchers.

A total of 480 questionnaires were distributed during the formal data collection phase, and all were returned. During data screening, 8 questionnaires were excluded based on data quality concerns, 3 questionnaires were excluded due to a substantial number of missing responses, and 5 questionnaires were excluded because the same response option was selected for substantive items. Therefore, 472 valid questionnaires were retained for the final analysis (valid response rate = 98.3%). As shown in Table 1, the final sample consisted of junior high school students with a nearly equal gender distribution (50.4% male and 49.6% female). Participants were recruited from Grades 7, 8, and 9, with similar percentages in each grade (33.5%, 33.7%, and 32.8%). Furthermore, the age composition varied between 12 and 15 years, with 13 years (32.0%) and 14 years (32.8%) showing the highest percentages, followed by 12 years (18.4%) and 15 years (16.7%). Overall, the sample showed a relatively balanced distribution across the main demographic characteristics.

### 2.2. Measures

A composite questionnaire was used to collect data. The questionnaire consisted of five scales measuring ASE, PPC, and PEI, namely, the Academic Self-Efficacy Scale, Father’s Psychological Capital Scale, Mother’s Psychological Capital Scale, Father’s Educational Involvement Scale, and Mother’s Educational Involvement Scale. All items were presented in Chinese. Although the Academic Self-Efficacy and Parental Educational Involvement Scales were originally developed by Chinese scholars, all instruments were carefully reviewed and adapted to ensure their suitability for the current study context. As all measures were completed by students, particular attention was given to the clarity and appropriateness of item wording from the perspective of junior high school students. A translation and back-translation procedure was conducted where necessary. In addition, experts in educational psychology reviewed all instruments to evaluate item clarity, cultural appropriateness, and suitability for junior high school students. Necessary revisions were made based on the experts’ feedback before the pilot study.

A pilot study was conducted with 104 junior high school students who were not included in the main sample. Exploratory factor analysis (EFA) was performed to examine the factor structures of the adapted instruments and assess the suitability of individual items. The evaluation of the EFA results followed established criteria. Specifically, items with factor loadings below 0.40, communalities below 0.40, or substantial cross-loadings were considered inadequate and subject to removal. Items meeting these criteria were retained to ensure that they adequately represented the intended constructs. The scales are described in detail below.

#### 2.2.1. Academic Self-Efficacy Scale (ASES)

The Academic Self-Efficacy Scale consists of 22 items divided into two dimensions: Academic Self-Efficacy in Learning Ability (ASELA) and Academic Self-Efficacy in Learning Behavior (ASELB) (Liang, 2000). Responses are rated on a 5-point Likert scale. Example items from the scale include “I believe I have the ability to do well in my studies” and “I always highlight key sections in my books or notebooks to help with studying”. In the pilot study, exploratory factor analysis resulted in the removal of 4 items, reducing the scale to 18 items. In this study, the ASES demonstrated good construct validity (χ^2^/df = 2.262, CFI = 0.955, TLI = 0.949, RMSEA = 0.059) and excellent reliability (Cronbach’s *α* = 0.937).

#### 2.2.2. Parental Psychological Capital Questionnaire (PPCQ)

The Parental Psychological Capital Questionnaire (PPCQ) was adapted from the Psychological Capital Questionnaire (PCQ-24) developed by Luthans et al. (2007) to assess adolescents’ perceived parental psychological resources in the family education context. Two parallel versions were adapted for the current study to assess adolescents’ perceived psychological capital of their fathers and mothers: the Father’s Psychological Capital Questionnaire (FPCQ) and the Mother’s Psychological Capital Questionnaire (MPCQ). Both questionnaires assess four dimensions of psychological capital: self-efficacy, hope, resilience, and optimism. The original questionnaire consisted of 24 items rated on a five-point Likert scale ranging from 1 (almost never) to 5 (almost always). Following the pilot EFA, 5 items were removed from each parent version, resulting in 19 items on both the FPCQ and MPCQ. Example items include “My father believes he can analyze long-term problems and find solutions” and “My mother usually takes the stress of parenting in stride.” This study demonstrated an acceptable fit for the FPCQ (χ^2^/df = 2.250, CFI = 0.965, TLI = 0.960, RMSEA = 0.052) and MPCQ (χ^2^/df = 2.460, CFI = 0.953, TLI = 0.946, RMSEA = 0.056). Reliability analysis further demonstrated good internal consistency, with Cronbach’s alpha coefficients of 0.925 for the FPCQ and 0.923 for the MPCQ.

#### 2.2.3. Parental Educational Involvement Scale (PEIS)

The Parental Educational Involvement Scale was established by Song (2010) and contains two subscales: the Father’s Educational Involvement Scale (FEIS) and the Mother’s Educational Involvement Scale (MEIS). Each subscale of the PEIS measures three domains: emotional management, intellectual management, and behavioral management. Responses are scored on a 5-point scale ranging from 1 (almost never) to 5 (almost always). Following the pilot EFA, three items were removed from the FEIS, and two items were removed from the MEIS. Example items from the scale include “My father employs positive reinforcement techniques, such as verbal commendation, to foster my motivation for learning” and “My mother engages in communication with me regarding school events or activities that pique my interest.” This study demonstrated an acceptable fit for the FEIS (χ^2^/df = 2.128, CFI = 0.972, TLI = 0.968, RMSEA = 0.049) and MEIS (χ^2^/df = 2.061, CFI = 0.971, TLI = 0.967, RMSEA = 0.047). The Cronbach’s alpha values of the father and mother subscales used in this study were 0.941 and 0.940, respectively.

### 2.3. Data Analysis

Data were entered and organized using IBM SPSS Statistic 30.0, with missing values and outliers appropriately handled to maintain data integrity. Descriptive analysis of all variables was carried out after data cleaning to provide a summary of sample characteristics and assess the distribution of variables. To reduce the potential for method effects, common method bias (CMB) was tested. The evaluation of construct validity was based on several indices, such as the composite reliability (CR), average variance extracted (AVE), and standardized loading coefficient (SLC), as well as the Pearson correlation coefficient for the convergent and discriminant validity (Fornell & Larcker, 1981).

IBM SPSS AMOS 24.0 was employed to examine the hypothesized structural relationships, including direct and mediated effects. Model fit was evaluated using multiple indices, including the χ^2^/df ratio, comparative fit index (CFI), Tucker–Lewis index (TLI), RMSEA, and SRMR, following recommended criteria. Bootstrapping with 5000 resamples was conducted to test the significance of indirect effects and estimate confidence intervals for mediation pathways (Preacher & Hayes, 2008; Hayes, 2022). To compare paternal and maternal effects, father- and mother-related variables were included simultaneously in the corresponding models. Differences in direct effects were examined by comparing constrained and unconstrained models using chi-square difference tests (Δχ^2^) (Kline, 2023). Differences in indirect effects were assessed by comparing two indirect effects using bootstrapped confidence intervals, with a confidence interval excluding zero indicating a significant difference (Coutts & Hayes, 2022).

## 3. Results

### 3.1. Measurement Assessment and Validation

Harman’s single-factor method was employed to evaluate possible common method variance. The results showed that the first factor accounted for 20.248% of the total variance, indicating that no single factor explained the majority of the variance (Podsakoff et al., 2003). Therefore, common method variance is unlikely to be a major concern in the present study.

**Figure 2:**
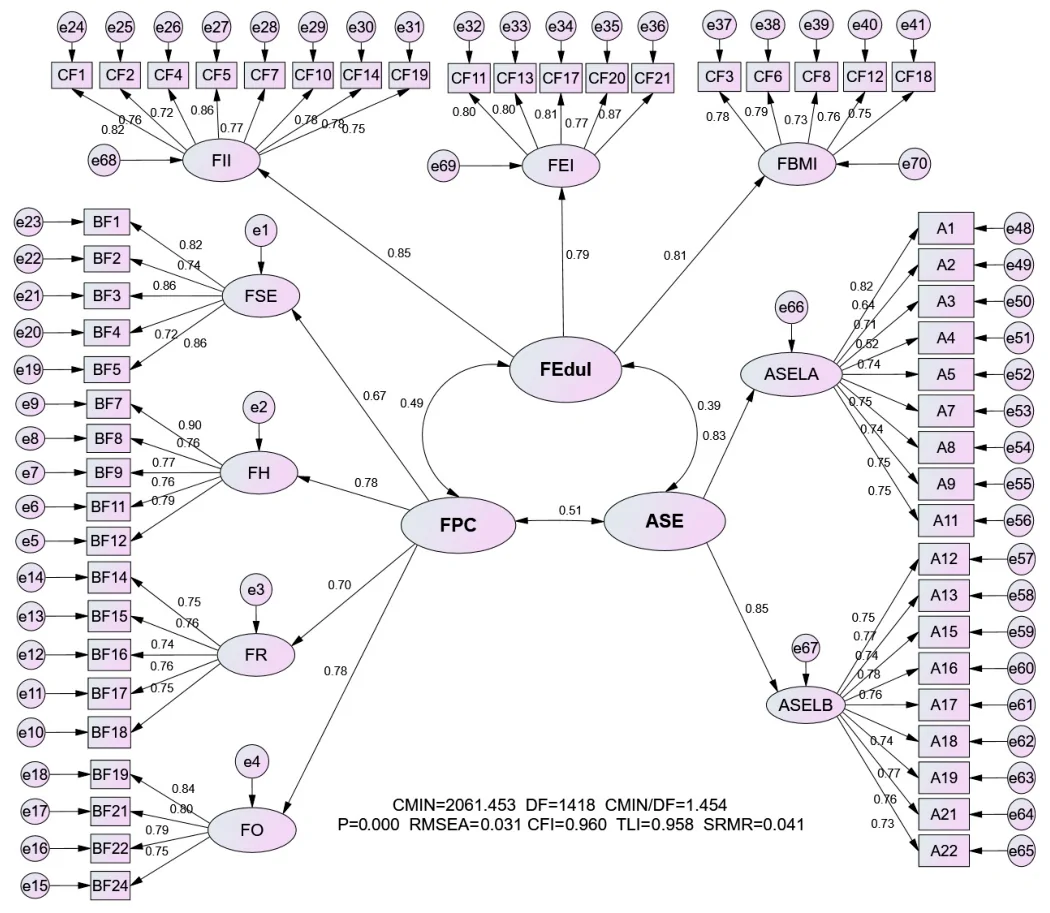
Father’s overall measurement model.

**Figure 3:**
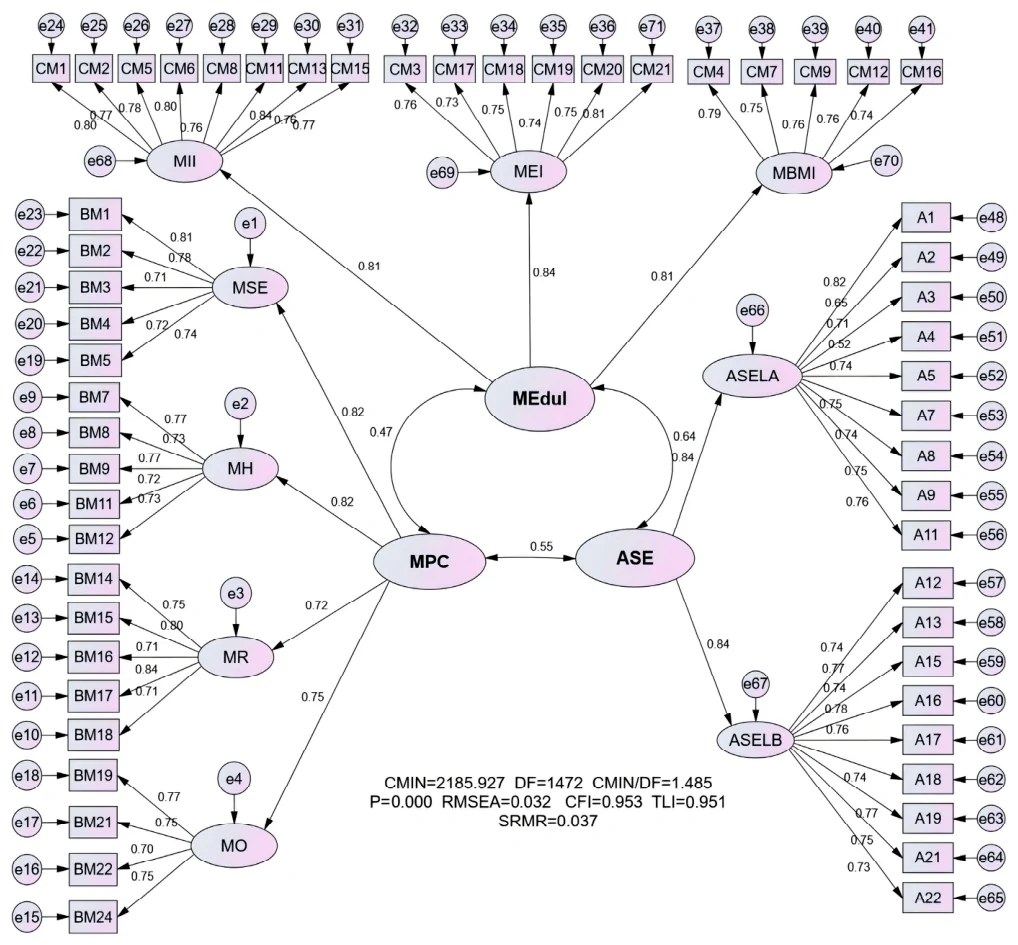
Mother’s overall measurement model.

The convergent and discriminant validity of the measures were explored to assess their construct validity. The standardized factor loadings, composite reliability values, and average variance extracted values were used to assess convergent validity. The factor loadings fell between 0.52 and 0.90, indicating acceptable item performance, as shown in Figure 2 and Figure 3. All latent variables’ composite reliability values exceeded the suggested 0.70 threshold (Hair et al., 2019), showing that internal consistency was satisfactory. Further, the AVE values were found to range from 0.543 to 0.619 (Table 2 and Table 3), which were above the minimum value of 0.50 recommended by Kline (2023). The outcomes of this investigation provide evidence for convergent validity. The Fornell and Larcker (1981) criterion was used to assess discriminant validity. The square root of the AVE for each construct was larger than that for the links between constructs, as shown in Table 2 and Table 3, which indicates sufficient differentiation among the latent variables.

### 3.2. Measurement Model Assessment

To test the measurement model, PPC, PEI, and ASE were specified as three latent variables and examined using confirmatory factor analysis. The results showed a good fit between the proposed measurement model and the observed data. Regarding the father model, the fit indices indicated satisfactory model fit, with values of χ^2^ = 2061.453, df = 1418, χ^2^/df = 1.454, CFI = 0.960, TLI = 0.958, RMSEA = 0.031 (95% CI [0.028, 0.034]), and SRMR = 0.041. Similarly, the mother model demonstrated a satisfactory fit, with χ^2^ = 2185.927, df = 1472, χ^2^/df = 1.485, CFI = 0.953, TLI = 0.951, RMSEA = 0.032 (95% CI [0.029, 0.035]), and SRMR = 0.037. As presented in Table 4, all fit indices were within the recommended ranges, providing evidence for an adequate measurement model. The final model’s standardized parameter estimates are displayed in Figure 2 and Figure 3.

### 3.3. Descriptive Results of Study Variables

Descriptive statistics were used to explore the students’ perceptions of ASE and family-related variables. A five-point Likert scale was used for all constructs, ranging from 1 (almost never) to 5 (almost always). According to the interpretation criteria proposed by Alkharusi (2022), mean scores ranging from 1.00 to 2.33 indicate a low level, those ranging from 2.34 to 3.67 indicate a moderate level, and those ranging from 3.68 to 5.00 indicate a high level of perception. Table 5 presents the descriptive statistics for each construct. The results showed that all variables had mean scores above 2.34, indicating at least a moderate level of perception among students. In particular, academic self-efficacy (ASE), perceived father’s psychological capital (FPC), perceived father’s educational involvement (FEduI), and perceived mother’s educational involvement (MEduI) were at a moderate level. Perceived mother’s psychological capital (MPC) reached a high level, suggesting that students perceived mother’s psychological resources as relatively higher.

### 3.4. Inferential Statistical Analysis Findings

#### 3.4.1. Path Analysis

**Figure 4:**
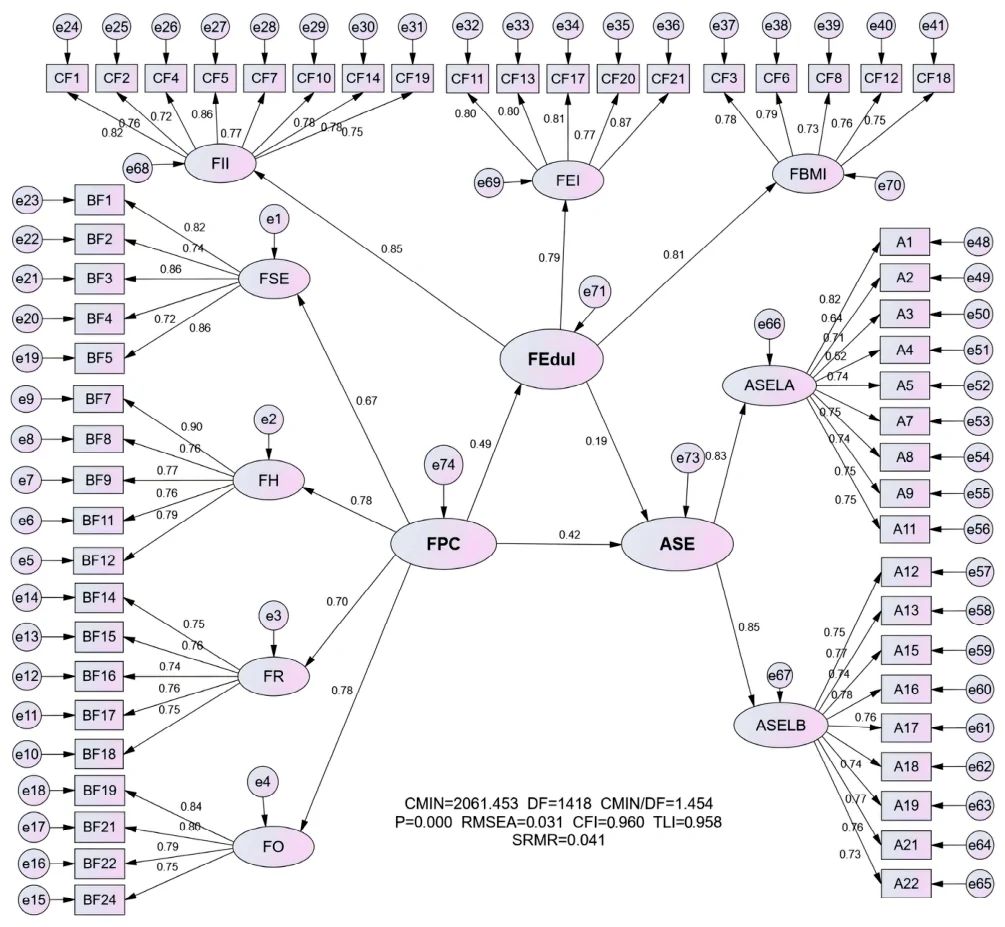
The standardized regression weights for each path in the model based on father’s data.

**Figure 5:**
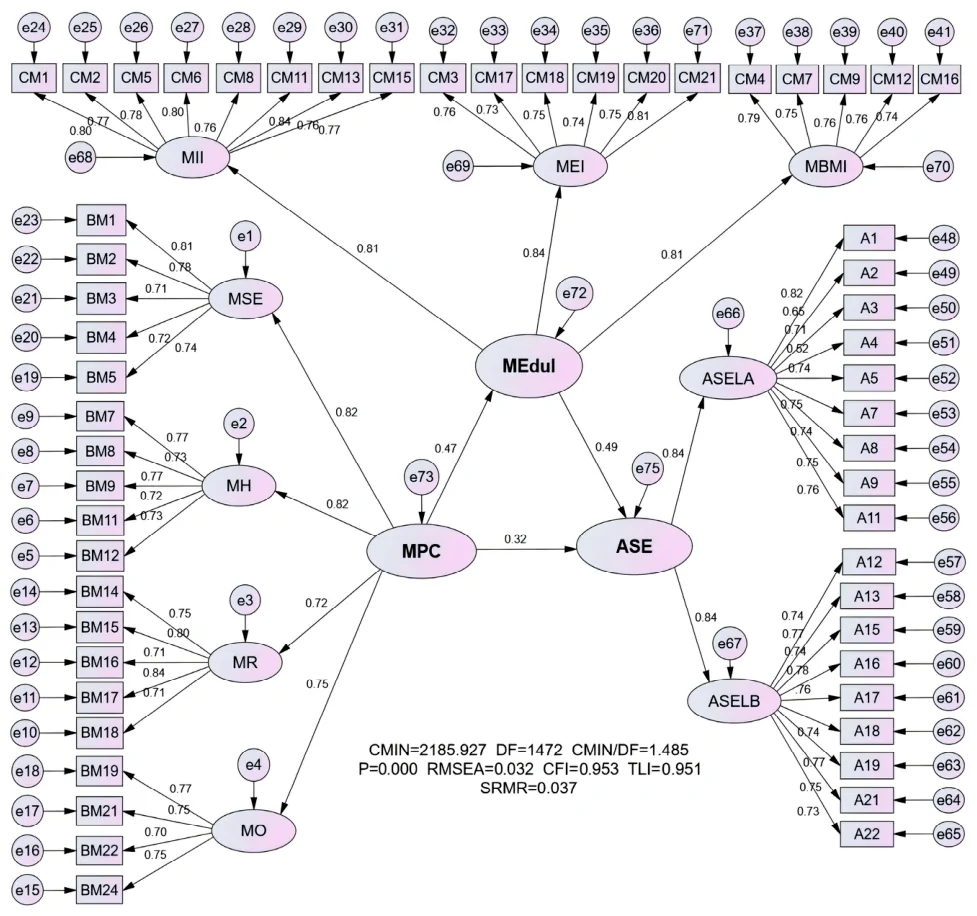
The standardized regression weights for each path in the model based on mother’s data.

Following confirmation of the satisfactory measurement model, structural equation modeling (SEM) was performed to test the hypothesized links between the latent variables. Models were tested separately for fathers (Model 1) and mothers (Model 2). The father model was found to be a good indicator of the data (χ^2^ = 2061.453, df = 1418, *p* < 0.001, χ^2^/df = 1.454, CFI = 0.960, TLI = 0.958, RMSEA = 0.031 (95% CI [0.028, 0.034]), and SRMR = 0.041). Likewise, the mother model also exhibited a good overall fit (χ^2^ = 2185.927, df = 1472, *p* < 0.001, χ^2^/df = 1.485, CFI = 0.953, TLI = 0.951, RMSEA = 0.032 (95% CI [0.029, 0.035]), and SRMR of 0.037) (see Figure 4 and Figure 5). The identical global fit indices observed for both the father and mother models reflect the fully recursive and saturated structural specification involving the three latent constructs. Overall, both structural models demonstrated a satisfactory fit to the observed data and were subsequently used to estimate and test the hypothesized structural relationships among the study variables.

The results of the structural path analysis are presented in Table 6. The findings showed that FPC significantly predicted students’ ASE (β = 0.419, *p* < 0.001); thus, H1a was supported. The effect of MPC on ASE (β = 0.321, *p* < 0.001) was statistically significant, supporting H1b. Regarding PEI, both FPC and MPC had significant positive effects on their respective educational involvement dimensions. Specifically, FPC significantly predicted FEduI (β = 0.490, *p* < 0.001), and MPC substantially predicted MEduI (β = 0.467, *p* < 0.001), supporting H2a and H2b. Furthermore, FEduI had a substantial positive effect on students’ ASE (β = 0.187, *p* = 0.004), and MEduI also had a substantial positive effect on ASE (β = 0.486, *p* < 0.001), supporting H3a and H3b.

These findings indicate that FPC and MPC were positively associated with students’ ASE and with their respective forms of PEI, while both forms of PEI were positively associated with ASE.

#### 3.4.2. Mediation Effects

In structural equation modeling, the assessment of mediating effects mainly focuses on the significance of the indirect effect, conceptualized as the multiplication of the path coefficients linking the independent variable to the mediator (a), and the mediator to the dependent variable (b). To assess the indirect effect, a bootstrap approach was used, which repeatedly resamples the data (e.g., 5000 bootstrap samples) to generate an empirical distribution of the indirect effect (Hayes, 2022; Preacher & Hayes, 2008). The mediation effect is deemed statistically significant when the 95% bootstrap confidence interval associated with the indirect effect excludes zero.

As shown in Table 7, 95% bias-corrected confidence intervals for the indirect effects did not include the value of zero, indicating that indirect effects were significant. In the father model, FPC had a positive direct path to ASE (B = 0.389, *p* < 0.001), as well as an additional indirect path through FEduI (B = 0.085, 95% CI [0.029, 0.156], *p* < 0.01). The total effect was 0.474, and the indirect effect through FEduI accounted for approximately 17.933% of the total effect (0.085/0.474). The same trend was observed in the mother model: There was a direct link between MPC and ASE (B = 0.304, *p* < 0.001), along with an indirect pathway via MEduI (B = 0.215, 95% CI [0.152, 0.304], *p* < 0.001). The total effect was 0.519, and the indirect effect through MEduI accounted for approximately 41.426% of the total effect (0.215/0.519).

Overall, these findings suggest that PEI serves as a significant indirect pathway linking PPC to students’ ASE, while direct effects of PPC remained significant in both models.

#### 3.4.3. Comparative Analysis of Direct Effect

To examine whether the structural paths differed significantly between the father and mother models, the pathways involving students’ perceived PPC and PEI were estimated simultaneously in a combined structural equation model. An unconstrained model was first estimated, in which the corresponding father and mother paths were freely estimated. Subsequently, equality constraints were imposed on the corresponding paths, and the constrained and unconstrained models were compared using the chi-square difference test (Δχ^2^).

As shown in Table 8, constraining the paths from students’ perceived PPC to ASE (FPC → ASE vs. MPC → ASE) to be equal did not significantly worsen model fit (Δχ^2^ = 0.285, Δdf = 1, *p* > 0.05), indicating that the two path coefficients did not differ significantly. In contrast, constraining the paths from students’ perceived PEI to ASE (FEduI → ASE vs. MEduI → ASE) resulted in a significant deterioration in model fit (Δχ^2^ = 12.874, Δdf = 1, *p* < 0.001), indicating a significant difference between the two pathways. As the path coefficient for MEduI → ASE was higher than that for FEduI → ASE, students’ perceived MEduI had a significantly stronger association with ASE than students’ perceived PEduI.

These findings indicate that the association between students’ perceived PPC and ASE did not differ significantly between fathers and mothers, whereas a significant father–mother difference was observed in the association between students’ perceived PEI and ASE. Therefore, H5a was partially supported.

#### 3.4.4. Comparative Analysis of Mediating Effect

The differences between the indirect effects of the paternal and maternal pathways were further examined using bootstrapped confidence intervals. As shown in Table 9, the indirect effect of perceived FPC on adolescents’ ASE through FEduI was 0.075 (95% CI [0.029, 0.156]). In comparison, the indirect effect of perceived MPC on ASE through MEduI was 0.197 (95% CI [0.152, 0.304]).

The difference between the two indirect effects was −0.123, with a 95% confidence interval ranging from −0.215 to −0.043. Because the confidence interval did not include zero, the indirect effects were found to differ significantly. Given that the indirect effect through MEduI was larger than that through FEduI, it was determined that the mediating role of MEduI was significantly stronger than that of FEduI. Therefore, H5b was supported.

## 4. Discussion

### 4.1. Relationship Between Students’ Perception of Parental Psychological Capital and Academic Self-Efficacy

The results of the present study revealed that students’ perceived PPC was significantly positively associated with their ASE, supporting H1a and H1b. The findings suggest that PPC may be perceived by adolescents as an important family-based psychological resource and is related to their confidence in handling academic problems. One possible explanation for the association between PPC and ASE may be understood from role modeling and observation–internalization perspectives. When parents demonstrate self-efficacy, hope, resilience, and optimism in the face of challenges, adolescents may perceive these qualities as psychological resources and incorporate similar qualities into their own academic experiences.

The overall results are consistent with those of previous research investigating the link between the psychological resources of parents and the outcomes of adolescents. Parental self-efficacy (part of psychological capital) has been found to be positively related to adolescents’ learning self-efficacy through supportive parenting practices (Holzer et al., 2024). Likewise, parents’ optimism and hope have been found to be associated with a positive emotional environment and more positive academic self-beliefs in adolescents (Pratt et al., 2001; Qi et al., 2022). More recently, researchers have begun to examine psychological capital as a holistic concept, and evidence suggests that maternal psychological capital is associated with parenting competence and child development outcomes (Yue et al., 2023; Yuan & Yue, 2022).

The results also underscore the need to examine the influence of family from the perspective of students. Adolescents’ views are crucial for understanding how perceived parental psychological resources are related to their ASE (De Los Reyes et al., 2019). These results indicate that interventions to enhance adolescents’ academic confidence should not be exclusively adolescent-centric. Supporting parents’ psychological resources, including self-efficacy, hope, optimism, and resilience, may represent a valuable direction for family-based programs aimed at promoting adolescents’ ASE. Workshops can be offered to schools or family programs, or resources can be provided to help parents learn these psychological skills. Such programs may also encourage parents to demonstrate goal-oriented behaviors and create a more supportive home environment for adolescents’ academic development.

### 4.2. Relationship Between Parental Psychological Capital and Parental Educational Involvement

The results of this study supported H2a and H2b, revealing that students’ perceived FPC and MPC were significantly and positively associated with their perceptions of FEduI and MEduI, respectively. These findings suggest that parents’ self-efficacy, resilience, hope, and optimism may represent important psychological resources associated with adolescents’ perceptions of parental roles in education.

Several cognitive, behavioral, and emotional perspectives may help explain the association between PPC and PEI. From a cognitive perspective, adolescents may evaluate their parents’ educational support based on their parents’ coping strategies and problem-solving abilities (Holzer et al., 2024; Lv et al., 2018). From a behavioral perspective, parents with higher psychological capital may be more likely to engage in behaviors such as regular monitoring, resource provision, and positive communication, which may be associated with adolescents’ perceptions of parental involvement (Gan & Bilige, 2019; Tazouti & Jarlégan, 2019). From an emotional perspective, parents’ positive emotions and expectations may be transmitted through emotional processes and contribute to a supportive family atmosphere, which may be related to adolescents’ perceptions of parental involvement (Kapetanovic & Skoog, 2021; Venard et al., 2024; Wang, 2018; Zhou et al., 2023).

These findings are consistent with those of previous studies that emphasize the significance of parental self-efficacy, hope, and optimism in education involvement (De Jong et al., 2022; Kashdan et al., 2002). While previous research has typically focused on one dimension of psychological capital, it is important to consider the higher-order construct of psychological capital as a whole to gain a deeper understanding of how parental psychological resources are associated with educational involvement.

### 4.3. Relationship Between Parental Educational Involvement and Academic Self-Efficacy

The present study found that junior high school students’ perceptions of FEduI and MEduI were significantly and positively associated with their ASE. These findings suggest that perceived PEI represents an important family-related resource associated with adolescents’ ASE.

Cognitive, behavioral, and emotional perspectives can help to understand this relationship. From a cognitive perspective, students evaluate their learning ability through interpretation of their parents’ guidance and feedback in learning activities (Fan & Williams, 2010). From a behavioral perspective, previous studies have suggested that mothers typically provide structured support and assistance in goal setting (Lv et al., 2018), while fathers tend to offer general encouragement and supervision (Kang et al., 2024). From an emotional perspective, supportive interactions may contribute to a positive atmosphere, which is associated with students’ sense of self-worth and confidence in learning (Gan & Bilige, 2019). These differences in parental roles may provide one possible explanation for why the association between MEduI and ASE was stronger than that between FEduI and ASE in the present study. However, this finding should not be interpreted as indicating that MEduI is inherently more important than FEduI. Rather, it may reflect adolescents’ perceptions of different patterns of parental engagement within the Chinese family context.

This finding is consistent with that of earlier investigations that found that PEI was positively associated with adolescents’ ASE (Fan & Williams, 2010; Gan & Bilige, 2019; Kang et al., 2024; Lv et al., 2018). However, some studies found that the overall effects of PEI are relatively small (Hill & Tyson, 2009). This discrepancy might be a result of the moderating effect of cultural context. Typically, parents in Chinese families place a high priority on their children’s education and play a significant role in helping their children manage their schoolwork and emotional issues in their daily lives (Chao, 2000; Tao & Lau, 2021). Furthermore, Cui et al. (2024) reported that over-engagement of parents can have a detrimental effect, and PEI may not be beneficial when it becomes excessive or controlling. The positive relationship identified in this study may indicate that adolescents perceived PEI as supportive rather than controlling.

Theoretically, the findings of the present study are consistent with social cognitive theory (Bandura, 1986). This theory provides a useful framework for understanding how adolescents may develop academic self-efficacy within the family context, particularly through observational learning and social interactions. To summarize, the findings of the present study show that perceived PEI is positively related to students’ ASE in the context of Chinese culture and provide implications for family-based education practices.

### 4.4. Mediating Role of Students’ Perceptions of Parental Educational Involvement in the Relationship Between Parental Psychological Capital and Their Academic Self-Efficacy

The present work revealed that the relationships between PPC and adolescents’ ASE were partially mediated through students’ perceptions of both FEduI and MEduI. Specifically, the results showed significant indirect associations between perceived PPC and ASE through perceived PEI, while direct associations between PPC and ASE remained significant. These indirect effects were supported by bootstrap confidence intervals.

The results are similar to those of previous studies demonstrating that parental psychological resources are associated with the outcomes of adolescents, as mediated by family processes (Lv et al., 2018; Yue et al., 2023). Previous research has often examined individual aspects of psychological capital, but this study considered self-efficacy, resilience, hope, and optimism as an integrated higher-order construct, providing a broader perspective for understanding how PPC may be related to adolescents’ academic development. Social cognitive and psychological capital theories provide useful theoretical perspectives for interpreting these findings, suggesting that parental psychological characteristics and related parenting behaviors may be perceived by adolescents through processes such as modeling, verbal encouragement, and daily interactions.

From a practical perspective, parents may benefit from opportunities to strengthen their psychological resources and develop supportive educational involvement strategies. Schools and family education programs can provide parent workshops and home–school collaboration platforms to promote parents’ awareness of psychological resources and supportive interactions with adolescents. These approaches may contribute to a more supportive family environment and provide adolescents with greater psychological resources for academic development.

### 4.5. Comparison of Two Separate Structural Equation Models Based on Students’ Perceptions of Father- and Mother-Related Variables to Examine Their Differential Effects

For the direct pathways examined in the comparative analysis, no significant difference was found between the father- and mother-related pathways from students’ perceived PPC to ASE. However, a significant difference was observed in the association between students’ perceived PEI and ASE, with the pathway from students’ perceived MEduI to ASE being stronger than that from students’ perceived FEduI to ASE. Furthermore, a comparison of indirect effects indicated that the indirect association between perceived PPC and ASE through MEduI was significantly stronger than that through FEduI. These findings suggest that, from the perspectives of adolescents in the present sample, MEduI may represent a more salient pathway associated with ASE than FEduI.

The stronger association between students’ perceived MEduI and ASE may be related to differences in the visibility and frequency of father- and mother-related educational involvement. Mothers are often more involved in daily learning supervision, homework assistance, and school-related communication, and these frequent educational interactions may provide adolescents with more opportunities to receive learning-related feedback and support. According to social cognitive theory (Bandura, 1997), mastery experiences, social persuasion, and supportive interactions are important sources of ASE. Therefore, adolescents may be more likely to associate maternal educational involvement with their own academic confidence because maternal involvement is often more directly experienced in daily learning contexts (Bandura, 1997; Fan & Williams, 2010). Meta-analytic evidence also indicates that, generally, the quality of MEduI is associated with greater autonomy-supportive practices (Xu et al., 2024). However, the stronger pathway observed for MEduI should not be interpreted as indicating that it is objectively more important than FEduI; rather, it may reflect adolescents’ perceptions of the forms of involvement that are more visible and frequent in their daily experiences.

This finding is somewhat different from that of previous studies reporting on the comparable effects of PEI, which may be attributed to methodological and contextual differences. Importantly, the present study examined adolescents’ perceptions of PPC and PEI rather than parents’ self-reported involvement or objectively observed behaviors. Therefore, the findings represent how adolescents perceive and interpret parental support, which may not completely correspond to parents’ actual behaviors or intentions. In the Chinese junior high school context, mothers may be more frequently perceived as involved in education because they often undertake more routine caregiving and learning-related responsibilities. These findings theoretically conflict with the assumption of parental uniformity and suggest that it is important to distinguish between the roles of parents in examining the family-based mechanisms that underlie ASE development.

The findings align with those of previous investigations that highlight the significance of the centrality of the mother in learning activities in different cultures (Fan & Williams, 2010; Grijalva-Quiñonez et al., 2020). Previous research has also suggested that mothers tend to report higher levels of involvement in their children’s educational activities, which may contribute to adolescents’ stronger perceptions of maternal involvement (Yue et al., 2023; Yuan & Yue, 2022). The differences between the present study and studies that found convergent parental effects (Liu et al., 2022) may be due to methodological differences, such as the use of student- versus parent-reported data (Kuhn et al., 2017).

These findings challenge the assumption that fathers and mothers contribute to adolescents’ academic development through identical pathways and suggest that parental roles may be perceived differently by adolescents within the family system. They also provide practical implications for family education. For mothers, interventions may focus on maintaining supportive, autonomy-promoting educational involvement, whereas fathers may be encouraged to increase their visible participation and provide motivational support during adolescents’ learning processes. Such differentiated approaches may facilitate complementary parental roles and contribute to more effective family education practices.

## 5. Limitations and Future Directions

This study has several limitations. First, the cross-sectional design limits the ability to draw clear causal inferences about the relationships among PPC, PEI, and adolescents’ ASE. Although the proposed model is theoretically grounded, future longitudinal or cross-lagged studies are needed to further examine the temporal relationships among these variables and improve our understanding of the intergenerational transfer of psychological resources in families. Second, potential common method bias should be considered. All variables in this study were assessed using self-report questionnaires at a single time point. Although Harman’s single-factor test indicated that common method variance is unlikely to be a major concern, this statistical approach has limitations and cannot completely rule out the possibility of bias. Future studies should adopt more rigorous approaches, such as the common latent factor method or marker variable technique, to better assess and address potential common method bias. Third, this study relied on the perspective of a single informant. All measures were obtained from students’ self-reports, which provided valuable insights into their perceptions of family experiences and academic development. However, relying solely on adolescents’ reports may limit a comprehensive understanding of the investigated constructs. Future research should incorporate multi-informant and mixed-method designs by including reports from parents and teachers, as well as observational or qualitative data, to further enhance construct validity. Fourth, the subjects of this research were junior high school students in China. Given that parental roles and educational involvement vary by culture, the generalizability of these findings may be context-specific. Replication of this study in other cultures and developmental stages would help to identify the boundaries of the proposed model and enhance its cross-cultural applicability. Finally, other contextual factors may impact the influence of PPC on adolescents but were under-investigated in this study as mediators. Future investigations will need to further extend the model by incorporating school- or student-level moderators in order to better understand the development of adolescents’ ASE.

## 6. Conclusions

The aims of this study were to examine the associations between PPC and junior high school students’ ASE, and to explore the mediating role of perceived PEI. According to the findings, students’ FPC and MPC were positively associated with their ASE, and these associations were partially mediated by perceived PEI. Furthermore, comparative analyses indicated that the association between perceived MEduI and adolescents’ ASE was stronger than that between perceived FEduI and adolescents’ ASE. In addition, the indirect association through perceived MEduI was significantly stronger than that through perceived FEduI, suggesting different perceived pathways linking FEduI and MEduI with adolescents’ ASE. The results indicate that parental psychological resources may function as important family resources associated with adolescents’ ASE while also being related to adolescents’ perceptions of PEI. In theoretical terms, this study contributes to psychological capital theory by providing evidence for how parental psychological resources are associated with adolescents’ academic self-efficacy within the context of family education. In practical terms, the findings highlight the importance of supporting parents’ psychological resources and promoting constructive educational involvement to foster adolescents’ confidence and motivation in school learning.

## Figures and Tables

**Table 1:** Respondents’ demographic profile (*N* = 472).

Personal Characteristic	Demographic Variable	Frequency (*n*)	Percentage (%)
Gender	Male	238	50.4
	Female	234	49.6
Grade	Grade 1(7)	158	33.5
	Grade 2 (8)	159	33.7
	Grade 3 (9)	155	32.8
Age	12	87	18.4
	13	151	32
	14	155	32.8
	15	79	16.7

**Table 2:** Results of correlation analysis, AVE, and CR (father’s variables).

Variable	Correlation Analysis, AVE, and CR				
	AVE	CR	1	2	3
Academic Self-Efficacy	0.543	0.956	1		
Father’s Psychological Capital	0.619	0.969	0.416 **	1	
Father’s Educational Involvement	0.614	0.966	0.315 **	0.402 **	1
AVE Mean Root Value			0.737	0.787	0.784

Note: AVE: average variance extracted; CR: composite reliability; ** *p* < 0.01.

**Table 3:** Results of correlation analysis, AVE, and CR (mother’s variables).

Variable	Correlation Analysis, AVE, and CR				
	AVE	CR	1	2	3
Academic Self-Efficacy	0.543	0.956	1		
Mother’s Psychological Capital	0.565	0.961	0.439 **	1	
Mother’s Educational Involvement	0.614	0.966	0.517 **	0.381 **	1
AVE Mean Root Value			0.737	0.752	0.784

Note: AVE: average variance extracted; CR: composite reliability; ** *p* < 0.01.

**Table 4:** Standards and thresholds for key metrics in CFA.

Fit Index	Abbreviation	Ideal Value/Threshold	Key Source(s)
Normed Chi-Square	χ^2^/df	<3 (good), <5 (acceptable)	Kline (2023)
Comparative Fit Index	CFI	≥0.95 (good), ≥0.90 (acceptable)	Hu and Bentler (1999)
Tucker–Lewis Index	TLI	≥0.95 (good), ≥0.90 (acceptable)	Hu and Bentler (1999)
Root Mean Square Error of Approximation	RMSEA	<0.05 (good), <0.08 (acceptable)	Steiger (1990); MacCallum et al. (1996)
Standardized Root Mean Square Residual	SRMR	<0.08 (good), <0.10 (acceptable)	Hu and Bentler (1999)

**Table 5:** Descriptive statistics for each construct.

	*n*	Minimum	Maximum	Mean	Std. Deviation
Academic Self-Efficacy	472	1.889	4.833	3.447	0.689
Father’s Psychological Capital	472	1.947	4.895	3.632	0.703
Father’s Educational Involvement	472	1.611	4.944	3.588	0.848
Mother’s Psychological Capital	472	1.895	4.947	3.684	0.717
Mother’s Educational Involvement	472	1.737	5.000	3.606	0.900
Valid N	472				

**Table 6:** Results of hypothesis testing using structural equation modeling.

Hypothesis	Hypothesized Path	B	S.E.	C.R.	*p*	β	Result
H1a	FPC → ASE	0.389	0.069	5.667	***	0.419	Supported
H1b	MPC → ASE	0.304	0.058	5.248	***	0.321	Supported
H2a	FPC → FEduI	0.589	0.078	7.523	***	0.490	Supported
H2b	MPC → MEduI	0.452	0.061	7.369	***	0.467	Supported
H3a	FEduI → ASE	0.145	0.050	2.892	**	0.187	Supported
H3b	MEduI → ASE	0.476	0.065	7.366	***	0.486	Supported

Note: ASE: academic self-efficacy; FPC: father’s psychological capital; MPC: mother’s psychological capital; FEduI: father’s educational involvement; MEduI: mother’s educational involvement; B: unstandardized regression coefficient; S.E.: standard error; C.R.: critical ratio; β: standardized regression coefficient; ** *p* < 0.01; *** *p* < 0.001.

**Table 7:** Bootstrap results for the mediating effect of PEI between PPC and ASE.

Path	Effect	B	*p* Value	Bootstrapping 95%	
				Lower Bound	Upper Bound
FPC → FEduI → ASE	Total Effect	0.474	***	0.367	0.592
	Direct Effect	0.389	***	0.272	0.514
	Indirect Effect	0.085	**	0.029	0.156
MPC → MEduI → ASE	Total Effect	0.519	***	0.405	0.642
	Direct Effect	0.304	***	0.188	0.425
	Indirect Effect	0.215	***	0.152	0.304

Note: ASE: academic self-efficacy; FPC: father’s psychological capital; MPC: mother’s psychological capital; FEduI: father’s educational involvement; MEduI: mother’ s educational involvement; B: unstandardized regression coefficient; ** *p* < 0.01; *** *p* < 0.001.

**Table 8:** Chi-square difference tests for father–mother differences in direct effects.

Path	Constrained Model		Unconstrained Model		Δχ^2^	Δdf	*p*	Path Difference
	χ^2^	df	χ^2^	df				
FPC → ASE vs. MPC → ASE	2202.439	1472	2202.154	1471	0.285	1	0.593	Not significant
FEduI → ASE vs. MEduI → ASE	1924.442	1420	1911.568	1419	12.874	1	<0.001	Significant

Note: FPC: father’s psychological capital; MPC: mother’s psychological capital; FEduI: father’s educational involvement; MEduI: mother’s educational involvement; Δχ^2^: chi-square difference between the constrained and unconstrained models; Δdf: difference in degrees of freedom.

**Table 9:** Comparison of indirect effects between father and mother pathways using bootstrapped confidence intervals.

Indirect Mediation Path	Estimate	95% CI Lower	95% CI Upper	Result
FPC → FEduI → ASE	0.075	0.029	0.156	
MPC → MEduI → ASE	0.197	0.152	0.304	
Difference	−0.123	−0.215	−0.043	Supported

Note: Estimates: unstandardized indirect effects (B). Difference: calculated as the indirect effect of the father pathway minus that of the mother pathway. A 95% confidence interval excluding zero indicates a significant difference between the two indirect effects.

## Data Availability

Data will be made available upon reasonable request to the corresponding author.

## Abbreviations

The following abbreviations are used in this manuscript:

ASE	Academic Self-Efficacy
ASES	Academic Self-Efficacy Scale
FBMI	Father’s Behavior Management Involvement
FEduI	Father’s Educational Involvement
FEI	Father’s Emotional Involvement
FEIS	Father’s Educational Involvement Scale
FH	Father’s Hope
FO	Father’s Optimistic
FPC	Father’s Psychological Capital
FPCQ	Father’s Psychological Capital Questionnaire
FSE	Father’s Self-Efficacy
FR	Father’s Resilience
FII	Father’s Intellectual Involvement
ASELA	Academic Self-Efficacy in Learning Ability
ASELB	Academic Self-Efficacy in Learning Behavior
LASE	Learning Ability Self-Efficacy
LBSE	Learning Behavior Self-Efficacy
MBMI	Mother’s Behavior Management Involvement
MEduI	Mother’s Educational Involvement
MEI	Mother’s Emotional Involvement
MEIS	Mother’s Educational Involvement Scale
MH	Mother’s Hope
MII	Mother’s Intellectual Involvement
MO	Mother’s Optimistic
MPC	Mother’s Psychological Capital
MPCQ	Mother’s Psychological Capital Questionnaire
MR	Mother’s Resilience
PEI	Parental Educational Involvement
PEIS	Parental Educational Involvement Scale
PPC	Parental Psychological Capital
PPCQ	Parental Psychological Capital Questionnaire
